# West Nile fever outbreak, Israel, 2000: epidemiologic aspects.

**DOI:** 10.3201/eid0704.010416

**Published:** 2001

**Authors:** M Weinberger, S D Pitlik, D Gandacu, R Lang, F Nassar, D Ben David, E Rubinstein, A Izthaki, J Mishal, R Kitzes, Y Siegman-Igra, M Giladi, N Pick, E Mendelson, H Bin, T Shohat

**Affiliations:** Internal Medicine C & Infectious Diseases, Rabin Medical Center, Beilinson Campus, Petach-Tikva 49100, Israel. miriw@post.tau.ac.il

## Abstract

From August 1 to October 31, 2000, 417 cases of West Nile (WN) fever were serologically confirmed throughout Israel; 326 (78%) were hospitalized patients. Cases were distributed throughout the country; the highest incidence was in central Israel, the most populated part. Men and women were equally affected, and their mean age was 54+/-23.8 years (range 6 months to 95 years). Incidence per 1,000 population increased from 0.01 in the 1st decade of life to 0.87 in the 9th decade. There were 35 deaths (case-fatality rate 8.4%), all in patients >50 years of age. Age-specific case-fatality rate increased with age. Central nervous system involvement occurred in 170 (73%) of 233 hospitalized patients. The countrywide spread, number of hospitalizations, severity of the disease, and high death rate contrast with previously reported outbreaks in Israel.


### West Nile Fever Outbreak, Israel, 2000: Epidemiologic Aspects

**Miriam Weinberger,* Silvio D. Pitlik,* Dan Gandacu,† Ruth Lang,‡ Faris Nassar,§ Debora Ben David,¶ Ethan Rubinstein,¶ Avi Izthaki,# Joseph Mishal,** Ruth Kitzes,†† Yardena Siegman-Igra,‡‡ Michael Giladi,‡‡ Neora Pick,§§ Ella Mendelson,¶¶ Hanna Bin,¶¶Tamar Shohat,## and Michal Y. Chowers‡**

*Rabin Medical Center, Petach Tikva, Israel; †Israel Ministry of Health, Jerusalem, Israel; ‡Meir Medical Center, Kfar Sava, Israel; §Western Galilee Hospital, Nahariya, Israel; ¶The Chaim Sheba Medical Center, Tel Hashomer, Israel; #Assaf-Harofe Medical Center, Zrifin, Israel; **Barzilai Medical Center, Ashkelon, Israel; ††Carmel Medical Center, Haifa, Israel; ‡‡Tel-Aviv Sourasky Medical Center, Tel-Aviv, Israel; §§Bnei-Zion Medical Center, Haifa, Israel; ¶¶Central Virology Laboratory, Tel Hashomer, Israel; and ##Israeli Center for Disease Control, Tel Hashomer, Israel

From August 1 to October 31, 2000, 417 cases of West Nile (WN) fever were serologically confirmed throughout Israel; 326 (78%) were hospitalized patients. Cases were distributed throughout the country; the highest incidence was in central Israel, the most populated part. Men and women were equally affected, and their mean age was 54±23.8 years (range 6 months to 95 years). Incidence per 1,000 population increased from 0.01 in the 1st decade of life to 0.87 in the 9th decade. There were 35 deaths (case-fatality rate 8.4%), all in patients >50 years of age. Age-specific case-fatality rate increased with age. Central nervous system involvement occurred in 170 (73%) of 233 hospitalized patients. The countrywide spread, number of hospitalizations, severity of the disease, and high death rate contrast with previously reported outbreaks in Israel.

In early August 2000, infectious disease specialists in hospitals in central Israel noted an increasing number of elderly patients admitted for encephalitis. By mid-August, when several blood and cerebrospinal fluid (CSF) samples tested positive for antibodies for West Nile (WN) virus, an outbreak was suspected. Eventually a WN fever epidemic that affected the entire country was recognized.

This article outlines the epidemiologic aspects of this WN fever epidemic; Chowers et al. (1) details the clinical characteristics. These are the first in-depth descriptions of an outbreak caused by WN virus involving an entire country.

### Methods

Serologic studies from blood and CSF were performed by one facility, the Central Virology Laboratory, Public Health Services, Israel Ministry of Health, at the Chaim Sheba Medical Center, Tel Hashomer. From August 1 to October 31, 2000, this laboratory reported 417 serologically confirmed cases of WN fever to the Epidemiology Department of the Israel Ministry of Health. These patients are the focus of our study.

Basic demographic data, date of disease onset, and the patients' final outcome (alive or dead) were available from the files of the Department of Epidemiology, Israel Ministry of Health, and health district offices around the country. From early September 2000, infectious disease specialists in hospitals throughout Israel were asked to complete a detailed questionnaire on clinical, epidemiologic, and laboratory data for patients with WN virus at their hospitals. Information on the clinical presentation of the patients at ambulatory settings was obtained from the health district offices.

Data on the Israeli population by age group, geographic distribution, and type of locality were retrieved from the Israeli Central Bureau of Statistics (2). Israel is divided into six major geographic districts (Jerusalem, Northern, Haifa, Center, Tel-Aviv, Southern), as well as the Judea, Samaria, and Gaza areas. The permanently inhabited places in Israel are divided into urban (≥2,000 population) and rural (<2,000, even if not agricultural) localities.

Diagnosis of WN fever was based on immunoglobulin M (IgM)-capture enzyme-linked immunosorbent assay (MAC-ELISA) in serum or CSF samples of patients. The assay, which was developed in the Central Virology Laboratory during 1999-2000, includes the following steps: a) binding of anti-human IgM to the ELISA plate; b) incubation with the patient's serum or CSF sample (dilutions of 1:100 and 1:2,000 for serum and 1:10 and 1:100 for CSF); c) incubation with WN virus antigen prepared from Vero cells infected with an Israeli gull isolate from 1999 (Banet C, manuscript in prep.); d) incubation with a mouse anti-flavivirus monoclonal antibody (JCU/KUN/2B2, cat. no. 01-031-02, TropBio Pty Queensland, Australia); and e) incubation with peroxidase-conjugated goat anti-mouse antibody (cat. no. 115-035-071, Jackson ImmunoResearch Laboratories, West Grove, PA) and substrate. Cross-reactivity with other flaviviruses was not thoroughly evaluated; however, no other endemic cross-reacting flavivirus is known to infect humans in Israel. A

Address for correspondence: Miriam Weinberger, Internal Medicine C & Infectious Diseases, Rabin Medical Center, Beilinson Campus, Petach-Tikva 49100, Israel; fax: 972-3-9221605; e-mail: miriw@post.tau.ac.il patient with a positive MAC-ELISA from serum or CSF was considered to have a serologically confirmed case of WN fever.

Serologic studies for WN virus were not routine in Israel before 2000; this changed in early August 2000, when the first cases of WN fever were documented. Blood or CSF samples for serologic studies were submitted by primary physicians or infectious disease consultants caring for hospitalized patients, based on clinical suspicion. Although not encouraged by the Israeli health authorities, blood samples for serologic studies were also submitted by primary physicians caring for ambulatory patients.

Contingency tables were tested for statistical significance by the chi-square test. Continuous variables were tested by the Student *t* test. SAS software (SAS Inc., Cary, NC) was used for data analysis.

### Results

### Geographic Distribution

From August 1 to October 31, 2000, 417 cases of WN fever were confirmed by the Central Virology Laboratory. Patients were distributed throughout 113 localities in Israel. The most populated localities in the coastal plains of Israel were the most heavily affected, followed by the northern parts. The Negev and Jerusalem areas were the least heavily affected. No cases of WN virus fever were reported from the Judea, Samaria, and Gaza areas. Incidence rates by geographic district are given in Figure 1.

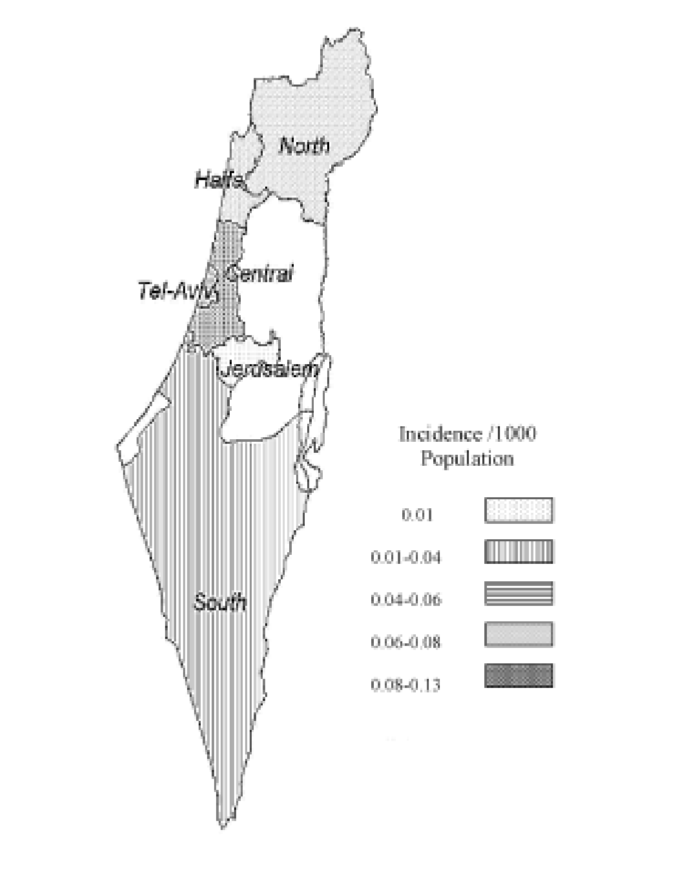

Figure 1. Incidence of West Nile fever infection by district, Israel, 2000.

Three hundred thirty-nine (81.5%) patients resided in urban localities, and 77 (18.5%) in rural localities; however, the incidence of WN virus infection was 2.3-fold higher in rural localities (0.14 cases per 1,000 population) than in urban areas (0.06 cases per 1,000 population, $p<0.001$).

### The Epidemic Curve

The exact date of disease onset was available for 379 (91%) patients with serologically confirmed WN fever. According to these data, the outbreak started at the end of July-early August, 2000, peaked in the second week of September, and abated by the end of October (Figure 2). The main wave of the epidemic started in the central parts of Israel (Center and Tel-Aviv districts), where 41% (2.5 million) of the Israeli population resides. One week later, the epidemic spread to the northern parts of Israel (Haifa and Northern districts), but peaked at the same time as in the central areas. A much smaller wave occurred in the south of Israel (Southern District): it started at the end of August and peaked at the end of September, 2 weeks later than the rest of the country (Figure 3).

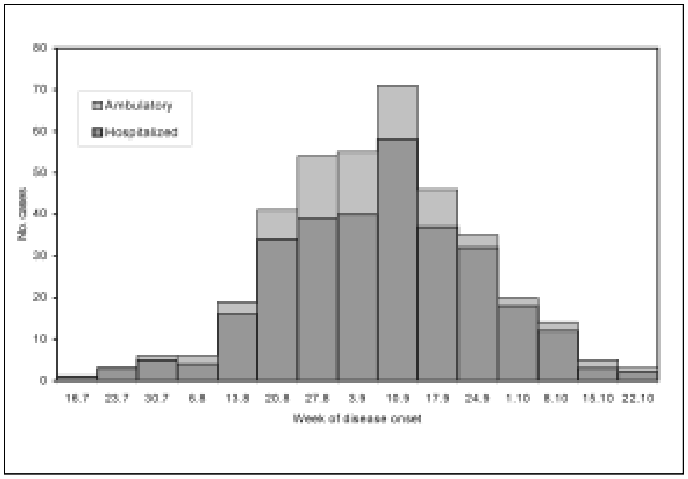

Figure 2. Serologically confirmed West Nile fever cases by week of disease onset, Israel, 2000.

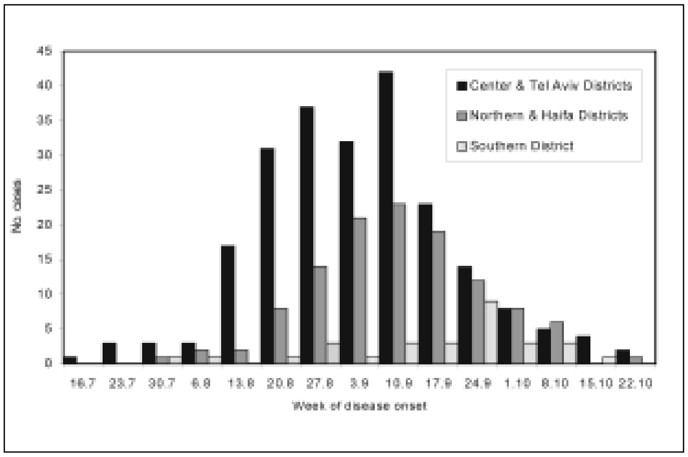

Figure 3. Distribution of West Nile fever cases by week of onset and geographic district, Israel, 2000.

### Patient Characteristics and Outcome

Of the 417 patients with serologically confirmed WN virus infection, 209 (50.1%) were female; age ranged from 6 months to 95 years (mean 54.5 ± 23.8 years, median 57). Only 6% (25 patients) were ≤14 years; 46% (192 patients) were ≥60 years. The overall incidence per 1,000 population was 0.07; incidence increased dramatically with age, from 0.01 in the 1st decade of life to 0.48 and 0.87 in the 9th and 10th decades, respectively (Figure 4). Notably, 15 (11.9%) of 126 hospitalized patients who were ≥60 years of age were residents of nursing homes.

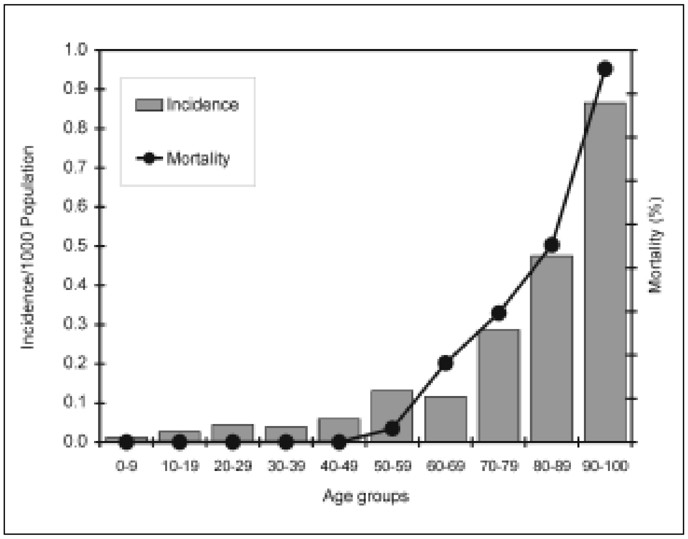

Figure 4. Incidence and deaths from West Nile fever by age group, Israel, 2000.

Three hundred twenty-six patients (78%) were hospitalized in 20 hospitals throughout Israel, and 91 (22%) were diagnosed in ambulatory settings. Ambulatory patients were significantly younger than hospitalized patients (mean age 44 ± 17.8 years vs. 57.4 ± 24.3 years, respectively, $p<0.0001$).

Clinical data were available for 233 (71%) of the 326 hospitalized patients and 37 (52%) of the 71 ambulatory patients. While 170 (73%) of the hospitalized patients had central nervous system (CNS) involvement (encephalitis 133 patients, meningitis 37), only 3 (8%) of the ambulatory patients had mild encephalitis. Further details are presented in a companion article (1).

Thirty-five (10.7%) of the 326 patients hospitalized with WN fever died during hospitalization (33 patients) or within 1 week after discharge (2 patients). None of the 91 ambulatory patients died. The case-fatality rate for all 417 patients was 8.4% (35 of 417 patients); this rate did not differ significantly between females (19 [9.1%] of 209) and males (16 [7.7%] of 208). The mean age of the patients who died was 79.1 ± 9.2 years (median 80), the youngest being 54 and the oldest 95 years old. In comparison, the mean age of the patients who survived was 52.2± 23.3 years ($p <0.0001$).

The case-fatality rate increased dramatically with age (Figure 4). Among all patients ages ≥60 years, the case-fatality rate was 17.7% (34 of 192). Predictors of death in hospitalized patients are discussed in a companion article (1).

### Discussion

WN fever is endemic in Israel (3-13); however, the impact of the 2000 outbreak was unprecedented. It involved all age groups, affected all parts of the country, resulted in the hospitalization of 326 patients within a 3-month period, and claimed the lives of 35 persons. The most prominent feature of this outbreak was an exceptionally high rate of CNS illness.

Israeli researchers in the 1950s were the first to characterize the clinical presentation of WN fever, but by the end of the 20th century, WN virus infection was an almost forgotten disease in Israel. Moreover, previous reports did not prepare the infectious disease community and health authorities for the scope, magnitude, and severity of the recent outbreak (14). Infectious disease experts quickly responded to the outbreak by arousing public opinion, enforcing preventive measures, exploring novel therapies (15, Huminer D, et al., unpub. data), and collaborating to form a detailed national clinical database.

Several WN fever outbreaks were reported from Israel in the 1950s and one in 1980 (Table 1) (4-12); most patients were young soldiers in training, who contracted the infection in army camps. Many of them were transferred to military hospitals in accordance with army routines (not necessarily because their illness was severe). The most common manifestation of WN fever in soldiers was an acute febrile illness with headache; a generalized rash occurred in approximately one third. CNS involvement was unusual, and the outcome was excellent.

Information about WN fever in the civilian population is more limited. In 1951, an outbreak occurred in a small agricultural settlement (Maayan Zvi) south of Haifa; 41% of its 303 inhabitants became ill (5,6). All age groups were affected, but only one child (0.8%) had mild aseptic meningitis, and no deaths were reported. Most adults in the settlement were 20 to 35 years old. Another report described a WN fever outbreak among 65 civilians residing in the Hadera area in 1957 (11); 2 patients (a child and an adult) had encephalitis (3.1%), which resolved without sequelae. No deaths were reported.

Standing out among these reports was a description of WN virus infection in 49 elderly patients, ages 66 to 86 years (mean 70 years), who were residents of four nursing homes in the Hadera area, the center of the 1957 epidemic (11). Encephalitis developed in one third of these patients and four died (8.1%). Serologic tests for WN virus were positive in 53% of the 49 patients (and 75% of those with encephalitis), and autopsy was compatible with encephalitis in one of four fatal cases. This was the first time that infection caused by WN virus was associated with severe encephalitis and death.

Some researchers believe (8,11) that a large outbreak of febrile illness accompanied by rash that occurred in 1941 (4), before the state of Israel was established, was also due to WN virus. Obviously, this could not be confirmed at the time, since the WN virus was a newly discovered virus (16) with unknown epidemiology, and diagnostic tests were not yet available. However, the clinical characteristics and time of year support the diagnosis of WN virus infection. An estimated 500 people in the Central and Tel-Aviv districts became ill. If one assumes that the diagnosis was correct, this was probably the largest outbreak in Israel, considering that the total population at that time was only 449,000 (2). In that outbreak no CNS involvement and no deaths were reported.

Table 1. Reported outbreaks caused by West Nile virus in Israel

| Year (Ref) | Area | Type of locality | No. studied | Age | CNS involvement | Reported deaths | Documentation |
|---|---|---|---|---|---|---|---|
| 1941 (4) | Center & Tel-Aviv | Urban & rural | 500 | All ages | None | None | None |
| 1951 (5,6) | Haifa | Agricultural settlement | 123 | All ages | Meningitis (1/123, 0.8%) | None | Virus isolate (1/123, 0.8%) Serology (14/26, 54%) |
| 1950 (7) | Hadera and coastal plain | Army camps and communal settlement | 105 | 17-40 y | Nuchal rigidity ("a few cases") | None | Virus isolate (9/50, 18%) Serology (18/24, 75%) |
| 1950-53 (8) | Military[a] hospital | Army camps | 400 | 17-23 y | Meningitis (1/400, 0.3%) | None | NA |
| 1953 (9) | Tel Hashomer Hospital[a] | Army camps | 70 | 18-20 y | None | None | Virus isolate (13/70, 18%) Serology (50/70, 68%) |
| 1953-1954 (10) | Center and North Israel | Army camps | 300 | Young adults | N/A | None | Virus isolate (40/205, 20%) Serology (66/151/ 57%) |
| 1957 (11) | Hadera area | Army camps | 300 | 18-28 y | Encephalitis (1/300, 0.3%) | None | Virus isolate (8/50, 40%) Serology (139/154, 88%) |
| | | Urban & rural | 65 | All ages | Encephalitis (2/56, 3.1%) | None | Serology (23/50, 46%) |
| | | Nursing homes | 49 | 66-86 y | Encephalitis 16/49 (33%) | 4/49, 8.2% | Serology (53%) (in patients with encephalitis: 9/12, 75%) |
| 1980 (12) | Negev Desert | Army camps | 32 | 18 y | Meningitis (1/32, 3.1%) Nuchal rigidity (3/32, 9.3%) | None | Serology (10/11, 91%) |
| 2000 (PR) | Country-wide | Urban and rural | 417 | 0.5-95 | Encephalitis (135/256, 51%) (35/265, 13%) | 35/417, 8.4% | Serology (417/417, 100%) |

[a]Soldiers were transferred to military hospitals according to army routines, not necessarily because of severe illness.
CNS = central nervous system; y = year; PR = present report.

The time of year in which WN fever outbreaks appear in Israel has remained almost constant for the last 5 decades. The previously reported outbreaks (4,6), as well as the recent one, occurred between late July and early October. In comparison, the 1996 epidemic in southeast Romania ended by mid-September (17), and the 1999 epidemic in New York abated by the end of September (18-20).

The severity and scope of the year 2000 outbreak in Israel can be better appreciated when compared with other WN virus outbreaks worldwide in the last decade (Table 2) (17,20-24). The Israeli outbreak most resembles the Romanian outbreak in 1996 (17), although both the incidence (7 vs. 4 per 100,000 population, respectively) and the death rate of hospitalized patients (10.7% vs 4.3%, respectively) were higher in Israel.

The vectors of WN virus in Israel are mosquitoes of the *Culex* species (*Cx. pipiens* and *Cx. perexiguus*) (25,26). The reservoir is wild birds, including pigeons, storks, and crows (25,27,28). In 1997, the virus infected the commercial geese population in Israel (Banet C, manuscript in prep.), a phenomenon that has not been previously described. This resulted in neurologic illness and death in the affected flocks. During 1998 and 1999, 18% to 20% of the 60 flocks in the country were affected, and approximately 5,000 geese died or were killed (O. Nir Markusfeld, pers. comm.). The virus was also isolated from live and dead horses (Banet C, manuscript in prep; 28).

Table 2. Reported deaths in WN virus outbreaks during the last decade

| Year | Location | No. cases studied | Deaths (%) | Reference |
|---|---|---|---|---|
| 1994 | Algeria | 13 | 13.3[a] | (21) |
| 1996 | South Romania | 393 | 4.3 | (17) |
| 1999 | New York | 61 | 11.5 | (20,22) |
| 1999 | South Russia | 1,000 | 4.0 | (23) |
| 2000 | New York, New Jersey | 19[b] | 10.5 | (24) |
| 2000 | Israel | 417 | 8.4 | Present report |

[a]Patients were mainly young children.
[b]Hospitalized patients only.

Human cases were not reported to the Ministry of Health during the 1990s until 1999, when three cases were diagnosed: a young woman with encephalitis who had fully recovered and two elderly patients who died of the disease (29,30). In late 1999 and the first quarter of 2000, the Central Virology Laboratory conducted a serologic survey in Eilot region, a rural area located in the Southern District. This survey revealed two additional cases of acute encephalitis and a 22% rate of IgM seropositivity to WN virus (E. Mendelson, pers. comm.).

A high degree of similarity (>99.8%) was found between the virus isolated from the brain of a dead Israeli goose in 1998 and the U.S. WN virus isolates from the 1999 epidemic (31). In the same study, an earlier Israeli WN virus isolate from 1952 was found to be phylogenetically closer to the 1996 Romanian isolate. More recent studies have also indicated that two phylogenetically distinct WN virus strains are cocirculating among humans (32) as well as avians and equines in Israel (Banet C, manuscript in prep.).

WN virus has reemerged in Israel with renewed virulence and vigor. One possible explanation is the waning of immunity among the Israeli population. Serologic studies from the 1950s in a small number of soldiers residing in epidemic areas revealed a 50% seropositivity during the outbreak season and a 14% seropositivity off-season (10). Other studies revealed 48% to 73% seropositivity in endemic areas, compared with 7% to 12% in nonendemic areas (13). The only large-scale study was published in 1999 by Cohen et al. (3), who tested stored sera of 1,060 soldiers, 18 to 55 years of age, which were collected between 1982 and 1989. Seroprevalence for WN virus was found to increase with age: from 7% in the 18- to 20-year age group to 10.5% in the 21- to 30-year age group, and as high as 41.9% for people ages 40 to 55. While revealing a high level of background immunity, these results do not support waning of seropositivity to WN virus with age.

The elderly population in Israel has almost doubled since the 1950s. In 1951, the proportion of patients $\geq$60 years was 6.8% (33); it increased to 13.2% in 1998, according to the most recent census (2). Forty-six percent of the WN fever cases and 97% of the fatal cases in the 2000 epidemic occurred in this age group. Unfortunately, no data are available on seroprevalence in the elderly in Israel. Pending results of a recently performed large serologic study in the general population, including nursing homes, may shed more light on this issue.

The changing face of WN virus epidemiology is not unique to Israel, but is a global phenomenon (34,35). Increased CNS invasiveness and a high case-fatality rate were also features of the outbreaks in southeast Romania and New York (17-20,36,37). This apparent increase in virulence had been also noted among birds in New York City (38) and commercial geese in Israel (Banet C, manuscript in prep.), who became sick and died as a result of WN infection. As more data are accumulating, it is tempting to speculate on a possible alteration in the virulence of WN virus. The altered strain may have been introduced into local avian populations by migratory birds, and then into humans and equines (Banet C, manuscript in prep.; 39).

The potential for causing large-scale outbreaks, with substantial illness and death, and the spread of WN virus across the globe call for international cooperation in developing effective vaccines and planning innovative strategies for mosquito control.

Dr. Weinberger is a senior infectious diseases consultant and head of the Infections in the Immunocompromised Host Service at the Rabin Medical Center, Petach-Tikva, Israel. Her main research interests include infections in the immunocompromised host and epidemiology and risk factors for fungal infections. Another focus of her research is emerging zoonoses, including salmonellosis and West Nile fever.

### References

1. Chowers MY, Lang R, Nassar F, Ben-David D, Rubinshtein E, Itzhaki A, et al. Clinical characteristics of West Nile fever outbreak, Israel, 2000. Emerg Infect Dis 2001;7:675-8.
2. Statistical abstracts of Israel. Jerusalem, Israel: Central Bureau of Statistics; 2000. Pub. no. 51.
3. Cohen D, Zaide Y, Karasenty E, Schwarz M, LeDuc JW, Slepon R, et al. Prevalence of antibodies to West Nile fever, sandfly fever Sicilian, and sandfly fever Naples viruses in healthy adults in Israel. Public Health Rev 1999;27:217-30.
4. Leffkowitz M. On the frequent appearance of an unclear infectious disease. Harefuah 1942;22:3-4.
5. Bernkopf H. The isolation of the West Nile virus in Israel. Harefuah 1953;45:99-101.
6. Bernkopf H, Levine S, Nerson R. Isolation of West Nile virus in Israel. J Infect Dis 1953;93:207-18.
7. Goldblum N, Sterk VV, Paderski B. West Nile fever. The clinical features of the disease and the isolation of West Nile virus from the blood of nine human cases. Am J Hyg 1954;59:89-103.
8. Radt P. Clinical observations on patients with West Nile fever during outbreaks of the disease in 1950-1953. Harefuah 1955;49:41-4.
9. Marberg K, Goldblum N, Sterk VV, Jasinska-Klingberg W, MA K. The natural history of West Nile fever. I. Clinical observations during an epidemic in Israel. Am J Hyg 1956;64:259-69.
10. Goldblum N, Sterk VV, Jasinska-Klingberg W. The natural history of West Nile fever. II. Virological findings and the development of homologous and heterologous antibodies in the West Nile infection in man. Am J Hyg 1957;66:363-80.
11. Spigland I, Jasinska-Klinberg W, Hofshi E, Goldblum N. Clinical and laboratory observations in an outbreak of West Nile fever in Israel. Harefuah 1958;54:275-81.
12. Katz G, Rannon L, Nili E, Danon YL. West Nile fever-occurrence in a new endemic site in the Negev. Isr J Med Sci 1989;25:39-41.
13. Flatau E, Kohn D, Daher O, Varsano N. West Nile fever encephalitis. Isr J Med Sci 1981;17:1057-9.
14. Siegel-Itzkovich J. Twelve die of West Nile virus in Israel. BMJ 2000;321:724.
15. Shimoni Z, Niven M, Pitlik D, Bulvik M. Treatment of West Nile virus encephalitis with intravenous immunoglobulin. Emerg Infect Dis 2001;7:759.
16. Smithburn KC, Hughes TP, Burke AW, Paul JH. A neurotropic virus isolated from the blood of a native of Uganda. Am J Trop Med Hyg 1940;20:471-92.
17. Tsai TF, Popovici F, Cernescu C, Campbell GL, Nedelcu NI. West Nile encephalitis epidemic in southeastern Romania. Lancet 1998;352:767-71.
18. Centers for Disease Control and Prevention. Outbreak of West Nile-like viral encephalitis—New York, 1999. MMWR Morb Mortal Wkly Rep 1999;48:845-9.
19. Centers for Disease Control and Prevention. Update: West Nile-like viral encephalitis—New York, 1999. MMWR Morb Mortal Wkly Rep 1999;48:890-2.
20. Centers for Disease Control and Prevention. Update: West Nile virus encephalitis—New York, 1999. MMWR Morb Mortal Wkly Rep 1999;48:944-6, 955.
21. Le Guenno B, Bougermouh A, Azzam T, Bouakaz R. West Nile: a deadly virus? Lancet 1996;348:1315.
22. Centers for Disease Control and Prevention. Guidelines for surveillance, prevention, and control of West Nile virus infection—United States. MMWR Morb Mortal Wkly Rep 2000;49:25-8.
23. Lvov DK, Butenko AM, Gromashevsky VL, Larichev VP, Gaidamovich SY, Vyshemirsky OI, et al. Isolation of two strains of West Nile virus during an outbreak in southern Russia, 1999. Emerg Infect Dis 2000;6:373-6.
24. Marfin AA, Petersen LR, Edison M, Miller J, Hadler J, Farrelo C, et al. Widespread West Nile virus activity, eastern United States, 1999-2000. Emerg Infect Dis 2001;7:730-5.
25. Nir Y, Avivi A, Lasovski Y, Margalit J, Goldwasser R. Arbovirus activity in Israel. Isr J Med Sci 1972;8:1695-701.
26. Samina I, Margalit J, Peleg J. Isolation of viruses from mosquitoes of the Negev, Israel. Trans R Soc Trop Med Hyg 1986;80:471-2.
27. Nir Y, Goldwasser R, Lasowski Y, Avivi A. Isolation of arboviruses from wild birds in Israel. Am J Epidemiol 1967;86:372-8.
28. Annual report. Beit-Dagan, Israel: The Israeli Veterinary Services; 2000.
29. Israel Ministry of Health. West Nile fever. Circular #44/2000 (September 19, 2000) 2000;2.
30. Giladi M, Metzkor-Cotter E, Martin DA, Siegman-Igra Y, Korczyn AD, Rosso R, et al. West Nile encephalitis in Israel, 1999: the New York connection. Emerg Infect Dis 2001;7:659-61.
31. Lanciotti RS, Roehrig JT, Deubel V, Smith J, Parker M, Steele K, et al. Origin of the West Nile virus responsible for an outbreak of encephalitis in the northeastern United States. Science 1999;286:2333-7.
32. Hindiyeh M, Shulman LM, Mendelson E, Grossman Z, Weiss L, Bin H. Isolation and characterization of West Nile virus from the blood of viremic patients during the 2000 outbreak in Israel. Emerg Infect Dis 2001;7:748-50.

33. Statistical abstracts of Israel. Jerusalem, Israel: Central Bureau of Statistics; 1958-59. Pub. no. 10.
34. Solomon T, Cardosa MJ. Emerging arboviral encephalitis. Newsworthy in the west but much more common in the east. BMJ 2000;321:1484-5.
35. Marra CM. Encephalitis in the 21st century. Semin Neurol 2000;20:323-7.
36. Sampson BA, Ambrosi C, Charlot A, Reiber K, Veress JF, Armbrustmacher V. The pathology of human West Nile virus infection. Hum Pathol 2000;31:527-31.
37. Shieh WJ, Guarner J, Layton M, Fine A, Miller J, Nash D, et al. The role of pathology in an investigation of an outbreak of West Nile encephalitis in New York, 1999. Emerg Infect Dis 2000;6:370-2.
38. Steele KE, Linn MJ, Schoepp RJ, Komar N, Geisbert TW, Manduca RM, et al. Pathology of fatal West Nile virus infections in native and exotic birds during the 1999 outbreak in New York City, New York. Vet Pathol 2000;37:208-24.
39. Rappole JH, Derrickson SR, Hubalek Z. Migratory birds and spread of West Nile virus in the Western Hemisphere. Emerg Infect Dis 2000;6:319-28.
